# Grass bud responses to fire in a semiarid savanna system

**DOI:** 10.1002/ece3.7516

**Published:** 2021-04-07

**Authors:** Quinn A. Hiers, Morgan L. Treadwell, Matthew B. Dickinson, Kathleen L. Kavanagh, Alexandra G. Lodge, Heath D. Starns, Doug R. Tolleson, Dirac Twidwell, Carissa L. Wonkka, William E. Rogers

**Affiliations:** ^1^ Department of Ecosystem Science and Management Texas A&M University College Station TX USA; ^2^ Department Rangeland, Wildlife, and Fisheries Management Texas A&M AgriLife Extension San Angelo TX USA; ^3^ US Forest Service Northern Research Station Delaware OH USA; ^4^ College of Forestry Oregon State University Corvallis OR USA; ^5^ Department of Agronomy and Horticulture University of Nebraska at Lincoln Lincoln NE USA; ^6^ Present address: USDA ARS Northern Plains Agricultural Research Lab Sidney MT USA

**Keywords:** bud dormancy, fire management, herbaceous perennial resprouting, plant mortality, vegetative tiller reproduction

## Abstract

Increasingly, land managers have attempted to use extreme prescribed fire as a method to address woody plant encroachment in savanna ecosystems. The effect that these fires have on herbaceous vegetation is poorly understood. We experimentally examined immediate (<24 hr) bud response of two dominant graminoids, a C_3_ caespitose grass, *Nassella leucotricha*, and a C_4_ stoloniferous grass, *Hilaria belangeri*, following fires of varying energy (J/m^2^) in a semiarid savanna in the Edwards Plateau ecoregion of Texas. Treatments included high‐ and low‐energy fires determined by contrasting fuel loading and a no burn (control) treatment. Belowground axillary buds were counted and their activities classified to determine immediate effects of fire energy on bud activity, dormancy, and mortality. High‐energy burns resulted in immediate mortality of *N. leucotricha* and *H. belangeri* buds (*p* < .05). Active buds decreased following high‐energy and low‐energy burns for both species (*p* < .05). In contrast, bud activity, dormancy, and mortality remained constant in the control. In the high‐energy treatment, 100% (*n* = 24) of *N. leucotricha* individuals resprouted while only 25% (*n* = 24) of *H. belangeri* individuals resprouted (*p* < .0001) 3 weeks following treatment application. Bud depths differed between species and may account for this divergence, with average bud depths for *N. leucotricha* 1.3 cm deeper than *H. belangeri* (*p* < .0001).

*Synthesis and applications:* Our results suggest that fire energy directly affects bud activity and mortality through soil heating for these two species. It is imperative to understand how fire energy impacts the bud banks of grasses to better predict grass response to increased use of extreme prescribed fire in land management.

## Graphical abstract

We experimentally examined the immediate (<24 hr) belowground bud responses of two dominant graminoids, a C_3_ caespitose grass, *Nassella leucotricha*, and a C_4_ stoloniferous grass, *Hilaria belangeri*, following fires of varying energy (J/m^2^) in a semiarid savanna in the Edwards Plateau ecoregion of Texas. Our results suggest that fire energy may directly affect bud activity and mortality for these two species. It is imperative to understand how fire energy impacts the bud banks of grasses to better predict grass response to increased use of extreme prescribed fire in land management.

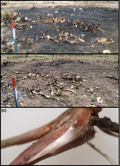

## 1 INTRODUCTION

In ecosystems dominated by perennial grasses, aboveground growth and persistence following disturbances are often determined by regrowth from a belowground bud bank (Dalgleish & Hartnett, 2009; Rogers & Hartnett, 2001). This type of growth is overwhelmingly prolific, with some research estimating that more than 99% of all new tiller growth originates from belowground buds (Benson & Hartnett, 2006). As opposed to seeds, these buried buds are associated with a parent plant and thus can remain dormant for a period of time and, once activated, the subsequent outgrowth is supported by the plant's resources (Ott et al., 2019). When disturbance frequency is intermediate, these populations of dormant buds are predicted to play a large role in the regeneration of many perennial grasses following disturbances such as herbivory or fire (Clarke et al., 2013) along with providing population stability in drought conditions (VanderWeide et al., 2014). This regeneration is likely constrained more by the rate of depletion and production of buds (bud bank size) rather than by the amount of resources available to support regeneration via buds (Cruz et al., 2003). Therefore, bud bank size not only determines the growth potential of perennial grasses, but can also directly determine a plant's ability to activate reserves, respond to disturbances, and react to pulses of high resource availability (Busso et al., 1989; Ott et al., 2019; Russell et al., 2015; VanderWeide et al., 2014).

Semiarid savannas developed under fire and grazing regimes that exerted selective pressures on plant community structure and composition (Milchunas & Lauenroth, 1993). In the presence of these aboveground disturbances, regrowth from a belowground bud bank that is insulated by a layer of soil offers a competitive advantage to herbaceous species that regenerate vegetatively from these buds (Dalgleish & Hartnett, 2009; Rogers & Hartnett, 2001; Russell et al., 2015). Fire suppression in turn has led to a marked increase in woody shrub encroachment into formerly herbaceous‐dominated plant communities (Twidwell et al., 2016; Archer et al., 2017).

Low‐energy fires have been utilized by land managers to sustain grass dominance as a substitute for the higher intensity fires that would naturally occur during the dry season. However, once invasion by woody species proceeds beyond a certain threshold, reintroducing low‐energy fire into the system is seldom a viable means to return to a grass‐dominated state (Ansley & Jacoby, 1998). Nevertheless, research has shown that high‐energy fires during drought can result in mortality of mature woody resprouting shrubs (Ansley & Jacoby, 1998; Twidwell et al., 2016).

Immediate changes in bud response to external factors such as disturbances may provide insight into possible long‐term fluctuations and structural shifts in plant community composition. Direct bud mortality due to disturbance may have a greater impact on species that maintain smaller bud banks and, in particular for C_3_ species, whose buds are short‐lived and recruit tillers from only the current year's buds (Ott & Hartnett, 2012). Increased bud death can also lead to meristem limitations, potentially resulting in a decreased future capacity to respond to external stimuli such as disturbances and changes in nutrient availability, light, and precipitation (Benson et al., 2004; Dalgleish & Hartnett, 2006; Ott et al., 2019). Overall, the bud bank size plays a fundamental role in local plant population structure and persistence and buffers against disturbance (Dalgleish & Hartnett, 2009), so short‐term decreases in bud numbers have the potential to alter population dynamics and community structure.

Prescribed fires, over the long term, maintain stable herbaceous community composition, especially when considering desirable grasses for rangeland managers (Taylor et al., 2012). When composition does change following fire, the primary drivers are legacy effects of pre‐existing variability rather than fire energy (Taylor et al., 2012). Although research has raised concerns that high‐energy fires may lead to long‐term negative effects in grass communities, little research has illustrated short‐term, immediate responses of grass bud banks to high‐energy fires.

The depth of the bud bank below the soil surface contributes to grass survival following disturbances, especially in the case of fire (Choczynska & Johnson, 2009). Soil is an insulator and retards the downward movement of heat into the soil (Clarke et al., 2013; Valettel et al., 1994). Although there are few relevant field studies that directly manipulate fire energy, we expect that high‐energy fires will result in greater heating at the soil surface (see Massman et al., 2010) as well as longer residence times of that heating due to high fuel loads. Studies have shown the impact of higher residence times on seed germination (Dayamba et al., 2010); it may be just as likely to have a significant effect on other plant tissues such as buds. As such, bud position in relation to the soil surface is important and most likely differs among grass growth forms. Stoloniferous and caespitose grasses typically have different bud depths due to their different vegetative growth strategies. Therefore, growth form traits and life‐history strategies likely drive differential effects of fire energy on the bud bank.

This study examined the effects of different levels of fire energy, achieved through two contrasting fuel loading treatments, on bud activity, dormancy, and mortality of two native perennial grass species with contrasting growth forms and photosynthetic pathways in a semiarid savanna during the summer dry season. The objectives of this study were to (a) assess the immediate (<24 hr following treatment) bud responses of a C_3_ caespitose grass and a C_4_ stoloniferous grass to different fire energies, (b) evaluate how bud depth may impact these bud responses, and (c) assess the impact of fire energy on initial reemergence of tillers for both species.

## 2 MATERIALS AND METHODS

### 2.1 Site description

Research was conducted at the Sonora Texas A&M Agrilife Research Station (SARS), which is on the western edge of the Edwards Plateau ecoregion in Texas (−100.574°, 30.251°). This semiarid, savanna experiences a bimodal precipitation pattern. The average annual precipitation varies from 356 to 889 mm, with the majority falling in the spring and fall. The average annual temperature ranges from 14 to 21°C, with summer temperatures reaching up to 41°C. The western Edwards Plateau historically experienced a fire return interval of 1–12 years, and fires were more common during late winter and late summer when grasses were dormant or dry and lightning strike frequency was high (Stambaugh et al., 2014).

The soils are in the Tarrant soil series (Clayey‐skeletal, smectitic, thermic Lithic Calciustolls; USDA, 2016), which tend to be very shallow and areas of exposed limestone bedrock are common. The dominant vegetation consists of a mosaic of trees and graminoids. The dominant trees in the area are *Quercus* spp., *Juniperus* spp., and *Prosopis glandulosa* Torr. The dominant graminoid species are *Hilaria belangeri* (Steud.) Nash, *Aristida* spp., *Bouteloua curtipendula* (Michx.) Torr., *Nassella leucotricha* (Trin. & Rupr.) R.W. Phol., and *Pleuraphis mutica* Buckley.

### 2.2 Experimental design and fire measurements

Fire treatments were arranged in a randomized design with three treatments (no burn, low fire energy, and high fire energy) replicated 12 times for a total of 36 experimental plots. Each plot was 100 m^2^ and centered on a mature (10+ years) mesquite shrub (*Prosopis glandulosa*) ranging from 3 to 5 m in height.

**FIGURE 1:**
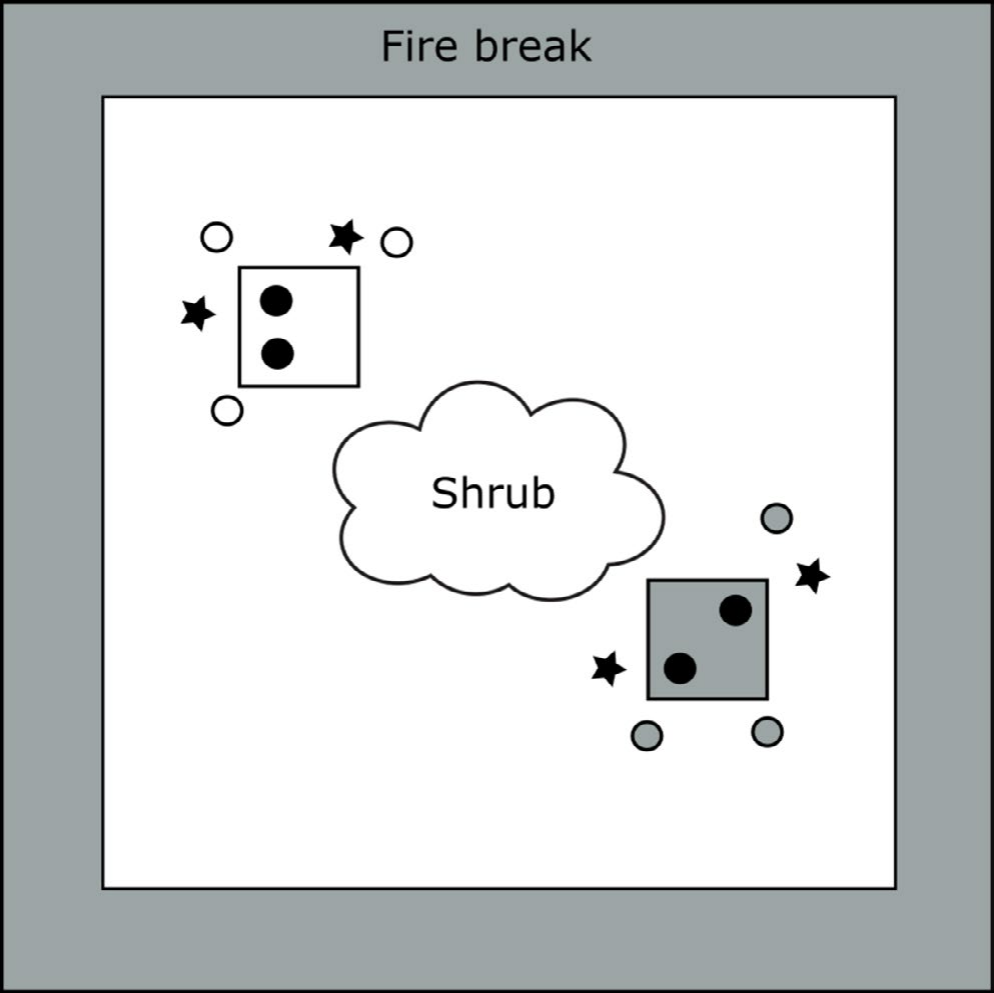
Visual representations of the main methodologies conducted in our experimental plots. All plots were 100 m^2^ and centered on a mature mesquite shrub, with a 1.8 m fire‐break around the periphery. The two smaller squares are an example of where subplots were created, one subplot per species in each plot. The black circles inside the subplots represent permanently marked individuals evaluated for regrowth 3 weeks following treatment application; circles outside subplots represent random individuals chosen for tiller collections and bud assessments. Black stars represent individuals chosen for bud bank depth measurements, 2 individuals per species in each plot

Two grass species, *N. leucotricha* and *H. belangeri*, were selected for this study due to their relative abundance at the site and for their contrasting phenological and growth form characteristics. Within each 10 × 10 m plot, two 1‐m^2^ subplots were demarcated with steel posts (Figure 1). One of these subplots was created around a patch of *N. leucotricha*, and the other around a patch of *H. belangeri*, and both served as a reference group for tiller collections described in the next section. Due to *H. belangeri's* stoloniferous growth form, we defined an individual as a single‐rooted node from which tillers arose. Within each of these subplots, two individuals of the focal species were marked and monitored for regrowth 3 weeks following fire application.

The last time our study site was burned was in August of 2000 with a high‐energy prescribed burn. Historically, our site was moderately grazed by sheep and goats. One growing season prior to our burns, the pasture containing our study site was rested and all domesticated grazing ceased throughout the study. However, our study site was still subject to herbivory from wildlife including *Odocoileus virginianus*, *Axis axis*, *Lepus californicus*, *Sylvilagus floridanus*, and a wide variety of invertebrates.

In early spring 2018, the entire pasture, with exception of our experimental plots, was burned to reduce surrounding fuel loads in preparation for our experimental fires. Each plot had a surrounding 1.8 m mineral soil fire‐break installed using heavy machinery.

There are critical fireline intensity thresholds required to induce mortality of woody species (Twidwell et al., 2009, 2013, 2016). Therefore, to manipulate the amount of heat produced by our fires, we added fuels in a way to match critical fireline intensity thresholds achieved in Twidwell et al. (2013). Prior to fuel application, we conducted a series of trials to determine the amount of fuel required to produce flame lengths similar to those observed in this previous research. Fireline intensity (kW/m), an estimate of heat flux along flame fronts, is defined by Byram as the product of fuel consumption, heat of combustion, and rate of spread (Byram, 1959). We could not replicate fire intensities because of the ignition method (see below), but we could replicate fuel loading and, thus, fire energy, which is attendant on fuel consumption (Kremens et al., 2012).

To provide a continuous fuel load across each burn plot, we spread a target amount of 60 kg of hay (approximately 0.6 kg/m^2^ at ambient moisture content) evenly across each 10 × 10 m low‐ and high‐energy plot. In addition to the hay and to produce flame lengths at the lower end of fires in Twidwell et al. (2013), we spread a target amount of 200 kg of previously harvested and dried juniper branches in a circular area ≤7 m in diameter (approximately 5.3 kg/m^2^ at ambient moisture content) on half of the plots. We centered the juniper fuels on the focal mesquite shrub to concentrate energy release in the plot interior. Subplots in these high‐energy plots were set up within this circle to ensure proper heat dosage. We determined fuel moistures (on a dry mass basis) for each fuel class from collections made at the time fuels were weighed prior to spreading them on plots and on burn days. We measured volumetric soil moisture at three locations in each plot immediately prior to ignition using an EXTECH MO750 Soil Moisture Meter. We report average wind speed and relative humidity in the 10 min leading up to fire ignition based on data from a portable weather station that we moved among plots.

Each plot was ignited with a ring fire method using two drip torches. Fine fuels were consumed almost completely on plots and were not re‐sampled after fire. However, there was sometimes woody material remaining on juniper addition plots which we collected and weighed to determine consumption from prefire loading.

Fire radiated energy (FRE, kJ/m^2^) and residence times (s) of fires at each subplot were estimated using 1 Hz imagery from a longwave infrared camera. Although we estimated FRE, we refer to it as fire energy above and hereafter for simplicity and in a relative sense with the understanding that FRE is a fraction (~20%) of total fire energy (Kremens et al., 2012). We used a FLIR SC660 and its internal calibration to produce sequences of radiometric (effective) pixel temperatures. We used the low‐temperature range (high gain) setting (up to 500°C) for the purpose of accurately monitoring mesquite stem temperatures. A boom lift was used to elevate the camera to an oblique perspective upwind of the plot. Oblique imagery was orthorectified using GDAL based on GPS positions of posts at the corners of each 10 × 10 m plot which were identified by use of aluminum targets with low emissivity that appear black in the infrared. Each orthorectified scene was re‐gridded to 1‐m^2^ pixels and the pixels which corresponded to each subplot were identified based on subplot locations, again determined by GPS. All GPS positions were corrected using data from a base station. Python scripting was used to run the GDAL orthorectification and re‐gridding and to calculate radiated power, radiated energy (kJ/m^2^), and residence times.

Radiometric pixel temperatures were converted to fire radiated power through the Stefan–Boltzmann equation and the blackbody assumption (O'Brien et al., 2016). FRE is the time integral of fire radiated power over the period described by the full‐width at one‐half of the maximum of radiated power (i.e., the width from before to after the maximum). We used a 450°C radiometric temperature threshold to indicate flame presence in the 1‐m^2^ pixel corresponding to each subplot and then estimated residence time by adding up the time steps during which temperature was greater than 450°C. We used 450°C because it was close to but below both the saturation and the Draper point (525°C) and roughly coincided with visible flaming and our expectations about residence times for hay‐only fires, expectations based on characteristics of fires in other fuel beds dominated by fine fuels (e.g., Bova & Dickinson, 2008; Butler et al., 2016). We were not able to estimate fireline intensities because, once flames converge, there is no spread in ring fires and fireline intensity loses its meaning.

### 2.3 Sampling

Tillers were harvested 24 hr before and after fires from three randomly determined individuals of each grass species inside each large plot (Figure 1). Tillers were collected less than 3.5 m from the central shrub to ensure the selection of individuals within the additional fuel loading area in the high‐energy treatment and ensure consistent sampling in the low‐energy and control treatments. These tillers were collected from individuals in similar phenological stages as the permanently marked individuals using the classification system of Moore et al. (1991). All collected tillers were from current year growth and all vegetative. Plants visibly damaged by herbivores, insects, or pathogens were excluded.

In each large plot, two tillers were harvested from each individual plant using a trowel to keep above and belowground structures intact. The buds associated with these tillers were counted and their activity classified as either active, dormant, or dead using the Tetrazolium and Evans Blue staining procedures established by Busso et al. (1989).

The day before the fire treatments were applied, bud depth was measured. Two random individuals of each species from each large plot were selected (Figure 1). Individuals were chosen based on similar size and phenological stage as our permanently marked individuals. A hole dug at the base of each individual grass exposed the deepest buds. We only wanted to examine belowground buds, so extra precaution was taken to only collect data from multitiller individuals (5+ tillers) of *H. belangeri* which, from pre‐experiment trials, usually indicated a deeper rooting individual and deeper buds.

Bud depth was recorded as the distance between the mineral soil surface and the base of each tiller (approximately at the beginning of the root system). Because our study site had low productivity and few trees, there was very little organic matter or duff on site. However, in the few cases where there was senesced plant material, it was swept away to access the mineral soil.

The area of differentiation between the tiller and root was used as an indicator of where the deepest buds would be located on each tiller. The buds of these species are small and often require a microscope to view. Additionally, since we only examined buds associated with tillers, these buds are often covered by the leaf sheath and are not easily identified in the field. However, previous tiller‐collection and bud‐counting trials revealed that, for both species, the buds begin to grow on the base of the tiller, right above the differentiation between tiller and root. Therefore, the area at the base of the tiller, where the root system begins, was used as a quick indicator of where the deepest buds were located.

### 2.4 Statistical analyses

Immediate bud response data were analyzed using analysis of variance (ANOVA) tests (Table 1). Data were analyzed by species using analysis of variance (MIXED procedure of SAS, Littell et al., 2006) in order to quantify bud bank response immediately before and after prescribed burns. The model included sampling period (pre‐ and postfire), burn treatment, and their interactions as fixed effects with plots as a random effect. Active, dormant, and dead buds were used as response variables, and the experimental unit was plot. Model assumptions for normality were tested using Shapiro–Wilk tests with the UNIVARIATE procedure of SAS and the normality hypothesis was not rejected for any of the data. Each experimental plot was analyzed for differences in bud responses before and after treatment application. Mean separations were determined with tests of pairwise comparisons using the Tukey–Kramer method following significant *F* tests on main effects or interactions. Statistical significance was declared at *p* < .05 for all tests.

**TABLE 1:** ANOVA table for total, active, dormant, and dead buds for *N. leucotricha* and *H. belangeri*

*N. leucotricha*												
Factors	Total			Active			Dormant			Dead		
Effect	*df*	*F* value	Pr > *F*	*df*	*F* value	Pr > *F*	*df*	*F* value	Pr > *F*	*df*	*F* value	Pr > *F*
Period	28	10.61	0.0029	28	11.91	0.0018	28	0.41	0.5268	28	3.77	0.0624
Energy	28	0.67	0.5186	28	3.86	0.0332	28	0.73	0.4931	28	2.38	0.1109
Period * Energy	28	0.82	0.4494	28	6.26	0.0057	28	0.66	0.5236	28	8.87	0.001

*H. belangeri*												
Factors	Total			Active			Dormant			Dead		
Effect	*df*	*F* value	Pr > *F*	*df*	*F* value	Pr > *F*	*df*	*F* value	Pr > *F*	*df*	*F* value	Pr > *F*
Period	29	34.53	<0.0001	29	69.24	<0.0001	29	0.81	0.376	29	23.63	<0.0001
Energy	29	0.2	0.8171	29	6.64	0.0042	29	1.79	0.1849	29	10.2	0.0004
Period * Energy	29	8.99	0.0009	29	18.4	<0.0001	29	0.35	0.7081	29	11.13	0.0003

Note

Effects consisted of sampling period (Period: pre‐ and postfire), fire energy (Energy), and their interaction (Period * Energy) as fixed effects. The analyses were done by species, with total, active, dormant, and dead buds as response variables.

Although we had 36 experimental plots, following treatment application infrared imaging revealed that some of our high‐energy subplots did not reach expected radiative energy output because they were outside the fuel addition area. Therefore, four subplots for both *N. leucotricha* and *H. belangeri* were removed from the analyses.

Bud depth data were analyzed using a Mann–Whitney *U* test to compare *N. leucotricha* to *H. belangeri*. Treatment effects on reemergence of our species were analyzed using Fisher's exact test (for *H. belangeri*) and a chi‐square test (for *N. leucotricha*). Statistical significance was set at *p* < .05.

## 3 RESULTS

### 3.1 Fire weather, fuels, and fire characteristics

Burns were completed over 5 days from 30 July to 4 August 2018. Wind speed, relative humidity, and soil and fuel moisture are summarized in Table 2. Existing grass and added hay and juniper fuel loadings on a dry basis, both prefire and consumed, are provided in Table 3. Average FRE and residence times for subplots are summarized in Table 4. Because the infrared imagery was saturated at a radiometric temperature of 500°C, subplot FRE values in Table 4 are underestimates. This is particularly the case for high‐energy fires (hay plus juniper) while low‐energy fires (hay only) were minimally saturated. Regardless, fire radiated energy in juniper addition plots was substantially greater than energy than in hay‐only plots (Table 4) primarily because residence times were 50 times longer than on low‐energy plots. Because of the underestimate on high‐energy plots, fire energy can be thought of as a relative index. Residence times were little affected by saturation given our use of a 450°C threshold to indicate visible flame presence.

**TABLE 2:** Fire weather and burn day fuel moisture at the plot level for low‐ and high‐energy treatments

Treatment	Wind (km/hr)	RH (%)	Soil moisture (%)	Moisture (%)				
				Grass	Hay	Juniper		
						Foliage	10 hr	100 hr
Low‐energy	8 (4–11)	27 (20–35)	13 (11–15)	9 (5–17)	4 (<1–10)	NA	NA	NA
High‐energy	9 (4–16)	28 (23–31)	13 (9–15)	7 (5–9)	4 (<1–8)	4 (1–5)	5 (3–8)	7 (5–7)

Note

Average wind and RH and their ranges (in parentheses) are for all plots in each treatment. Fuel moistures are on a dry mass basis and are averages (and ranges) of mean burn day values for a partial set of plots. Volumetric soil moistures are averages (and ranges) of mean burn day values for all plots. Plots including grass subplots described in this paper (*N* = 12 per treatment) are a subset of total plots in the larger study (*N* = 24) for which we report weather and fuel data here.

**TABLE 3:** Fuel bed heights and fuel additions and consumed loading at the plot level for low‐ and high‐energy treatments

Treatment	Fuel bed height (m)	Native herbaceous fuel loading (kg/m^2^)	Loading (kg/m^2^)			
			Prefire		Consumed	
			Hay	Juniper	Hay	Juniper
Low‐energy	0.21 (0.14–0.29)	0.10 (0.03)	0.54 (0.07)	NA	0.54 (0.07)^a^	NA
High‐energy	0.92 (0.55–1.49)	0.09 (0.03)	0.59 (0.14)	5.18 (0.81)	0.59 (0.14)^a^	5.05 (0.82)

Note

Hay was added to both low‐ and high‐energy plots to achieve continuous burns over the 10 × 10 m plot area while juniper was only added to high‐energy plots. For high‐energy fires, dried juniper was spread in a circular area (averaged 6.8 m diameter, range 5.8–8.1 m) centered on the focal mesquite shrub in ½ of the plots. Juniper was a mix of foliage and 1, 10, and 100‐hr size‐class woody material. All loadings (mean with standard deviation in parentheses) are on a dry mass basis and were determined for the measured areas over which fuels were spread. Plots including grass subplots described in this paper (*N* = 12 per treatment) are a subset of total plots in the larger study (*N* = 24 per treatment) for which we report weather and fuel data here.

^a^ Hay consumption was nearly complete for all plots, and postfire loading was not measured.

**TABLE 4:** Fire radiated energy and residence times for 1‐m^2^ pixels corresponding to subplot locations in the low‐ and high‐energy treatment plots

Treatment	Number of subplots	Energy (kJ/m^2^)^a^	Residence time (s)^b^
Low‐energy	22	423 ± 46	23 ± 3
High‐energy	14	23,434 ± 2,523	1,216 ± 133

Note

Reported are averages (and standard deviation) for the subplots in hay‐only plots (low‐energy) or plots to which both hay and juniper fuels were added (high‐energy). Some subplots in the high‐energy plots were outside of the juniper fuel addition area and are excluded here and from analyses (see Methods).

^a^ Energy of grass mini‐plot (1 m^2^), time integrated over the period in which fire radiated energy was greater than or equal to ½ of the maximum (full‐width at ½ maximum) radiation.

^b^ Residence time at grass plot calculated as the time steps for which the 1‐m^2^ pixel radiometric temperature was greater than a threshold of 450°C.

### 3.2 *Nassella leucotricha* dynamics

**FIGURE 2:**
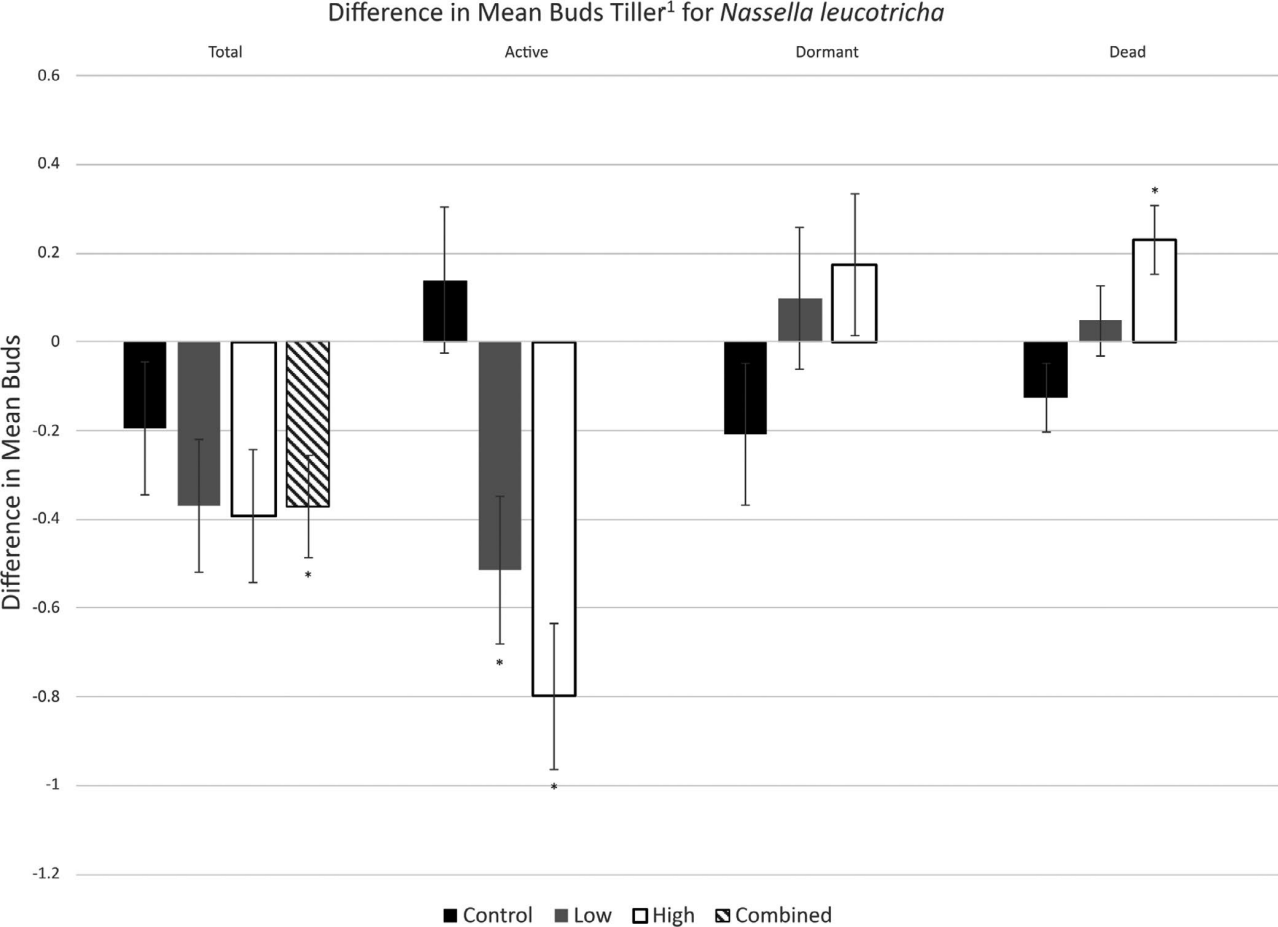
Difference in mean buds tiller^−1^ for *N. leucotricha* between pre‐ and post‐treatment values within each plot. Measurements were taken 24 hr before and after low‐ and high‐energy fire treatment application. Negative values indicate an average decrease in the number of buds tiller^−1^ from pre‐ to post‐treatment measurements within plots. Bars with an asterisk (*) indicate significant differences between pre‐ and post‐treatment values within treatment. For total buds, since only the period effect was significant, the treatments were combined and the overall change from pre‐ to post‐treatment was graphed. Pairwise comparisons were only performed when main or interaction effects were significant. Error bars indicate one standard error

Since only the period main effect was significant (Table 1), changes in total bud numbers were considered across all energy treatments. There was an immediate significant reduction in total buds for *N. leucotricha* between pre‐ and post‐treatment sampling times; total bud numbers across our treatments decreased by 17% (decrease of 0.37 ± 0.14 buds tiller^−1^; *t*_28_ = −2.53, *p* = .003; Figure 2; Table 5).

**TABLE 5:** Immediate (<24 hr) fire energy effects on the number of total, active, dormant, and dead belowground buds belonging to *N. leucotricha* and *H. belangeri* tillers

*N. leucotricha*						
Bud classification	Control		Low		High	
	Pre	Post	Pre	Post	Pre	Post
Total	2.3 ± 0.13	2.1 ± 0.13	2.2 ± 0.13a	1.9 ± 0.13ab	2.3 ± 0.16a	1.7 ± 0.16b
Active	0.9 ± 0.13ab	1.0 ± 0.13ab	1.2 ± 0.13a	0.7 ± 0.13b	1.0 ± 0.16ab	0.2 ± 0.16c
Dormant	1.2 ± 0.13a	0.99 ± 0.13a	1.0 ± 0.13a	1.1 ± 0.13a	1.3 ± 0.16a	1.2 ± 0.16a
Dead	0.18 ± 0.057a	0.056 ± 0.057a	0.083 ± 0.057a	0.13 ± 0.059a	0.083 ± 0.069a	0.42 ± 0.069b

*H. belangeri*						
Bud classification	Control		Low		High	
	Pre	Post	Pre	Post	Pre	Post
Total	2.8 ± 0.16a	2.8 ± 0.16a	3.3 ± 0.16b	2.2 ± 0.16c	3.3 ± 0.19b	2.0 ± 0.19c
Active	1.6 ± 0.16a	1.6 ± 0.16a	1.8 ± 0.16a	0.6 ± 0.16b	1.8 ± 0.19a	0.1 ± 0.19b
Dormant	1.2 ± 0.16a	1.2 ± 0.16a	1.5 ± 0.16a	1.5 ± 0.16a	1.6 ± 0.19a	1.3 ± 0.19a
Dead	0.014 ± 0.067a	0.014 ± 0.067a	0.014 ± 0.067a	0.18 ± 0.067a	0 ± 0.081a	0.69 ± 0.0.081c

Note

Pre‐ and post‐treatment values are given. All numbers represent bud means which are given in buds tiller^−1^. Means within bud classification are similar when followed by a common letter (*p* > .05). Pairwise comparisons were only performed when main or interaction effects were significant.

There was a significant effect of fire energy (Table 1) on the number of active buds for *N. leucotricha*. When comparing pre‐ and post‐treatment values, active bud numbers in the high‐energy fire treatment decreased by 84% (decrease of 0.79 ± 0.22 buds tiller^−1^; *t*_28_ = −3.68, *p* = .001; Figure 2; Table 5) while active bud numbers in the low‐energy treatment decreased by 42% (decrease of 0.49 ± 0.18 buds tiller^−1^; *t*_28_ = −2.72, *p* = .01; Figure 2; Table 5). Overall, our fire treatments significantly decreased active buds while the control treatment was unaffected. Twenty‐four hours following fire treatments, the total number of active buds for *N. leucotricha* differed between the low‐energy and high‐energy treatments. The low‐energy treatment had 0.52 ± 0.24 more buds tiller^−1^ than the high‐energy treatment post‐treatment (*t*_28_ = −2.41, *p* = .02).

There was no significant difference in dormant buds for *N. leucotricha* between pre‐ and post‐treatment sampling periods for any of our treatments (Table 1; Figure 2; Table 5).

There was a significant difference in dead buds between pre‐ and post‐treatment sampling periods for only the high‐energy treatment (Table 1). When comparing pre‐ and post‐treatment values, dead bud numbers in the high‐energy treatments increased by 424% (increase of 0.33 ± 0.08 buds tiller^−1^; *t*_28_ = 3.9, *p* < .001; Figure 2; Table 5).

### 3.3 *Hilaria belangeri* dynamics

**FIGURE 3:**
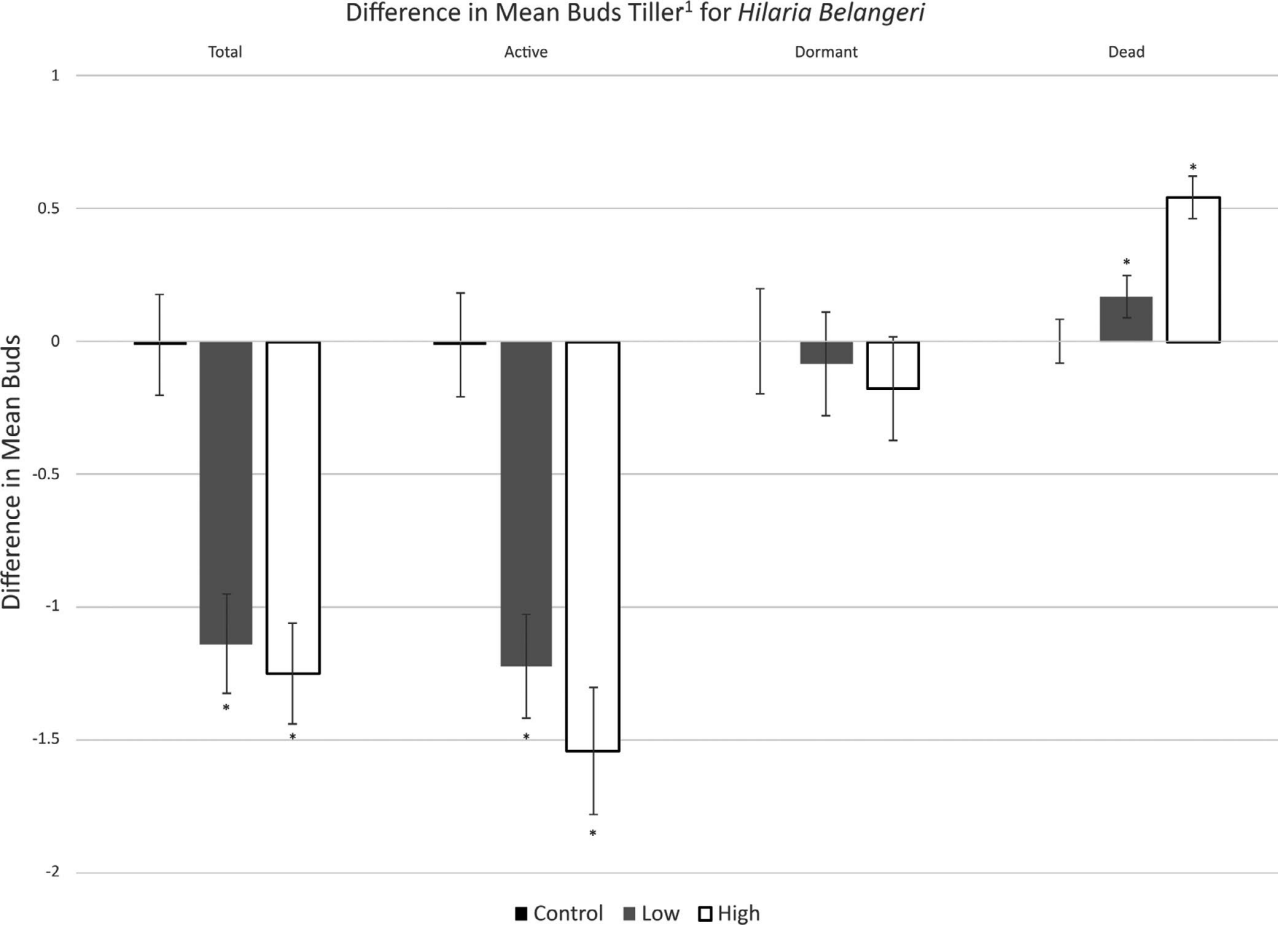
Difference in mean buds tiller^−1^ for *H. belangeri* between pre‐ and post‐treatment values. Measurements were taken 24 hr before and 24 hr after low‐ and high‐energy fire treatment application. Negative values indicate a decrease in the number of buds tiller^−1^ from pre‐ to post‐treatment measurements. Bars with an asterisk (*) indicate significant differences between pre‐ and post‐treatment values within treatment. Error bars indicate one standard error

There was a significant difference in total buds for *H. belangeri* between pre‐ and post‐ treatment sampling times for both the low‐ and high‐energy treatments but not the control (Table 1). When comparing pre‐ and post‐treatment values, total bud numbers in the high‐energy treatment decreased by 38% (decrease of 1.27 ± 0.27 buds tiller^−1^; *t*_29_ = −4.71, *p* < .001) while total buds in the low‐energy treatment decreased by 34% (decrease of 1.14 ± 0.22 buds tiller^−1^; *t*_29_ = −5.17, *p* < .001) following treatment application. Overall, fire treatments led to a significant decrease in the total number of *H. belangeri* buds (Figure 3; Table 5). However, the total number of buds post‐treatment was similar between the low‐energy and high‐energy treatments.

There was a significant difference in active buds for *H. belangeri* between pre‐ and post‐ treatment sampling periods for both the low‐ and high‐energy treatments but not the control (Table 1). When comparing pre‐ and post‐treatment values, active bud numbers in the high‐energy treatment decreased by 95% (decrease of 1.67 ± 0.23 buds tiller^−1^; *t*_29_ = −7.30, *p* < .001) while active buds in the low‐energy treatment decreased by 69% (decrease of 1.22 ± 0.19 buds tiller^−1^; *t*_29_ = −6.55, *p* < .001) following treatment. Overall, our fire treatments led to a decrease in the number of active *H. belangeri* buds (Figure 3; Table 5). However, the number of active buds post‐treatment was similar between the control, low‐energy, and high‐energy treatments.

There was no statistically significant difference in dormant buds between pre‐ and post‐treatment for any of the fire treatments (Table 1; Figure 3; Table 5).

There was a significant difference in dead buds for *H. belangeri* between pre‐ and post‐treatment for only the high‐energy treatments (Table 1). When comparing pre‐ and post‐treatment values, the number of dead buds in the high‐energy treatment increased by 0.69 ± 0.12 buds tiller^−1^; *t*_29_ = 5.98, *p* < .001).

### 3.4 Bud depths

Mean bud depth differed between *N. leucotricha* and *H. belangeri* (Mann–Whitney *U* = 2,720, *n*_1_ = *n*_2_ = 72, *p* < .001 two‐tailed). On average, bud depth was 1.8 ± 0.06 cm for *N. leucotricha* and 0.5 ± 0.04 cm for *H. belangeri*.

### 3.5 Initial reemergence

**FIGURE 4:**
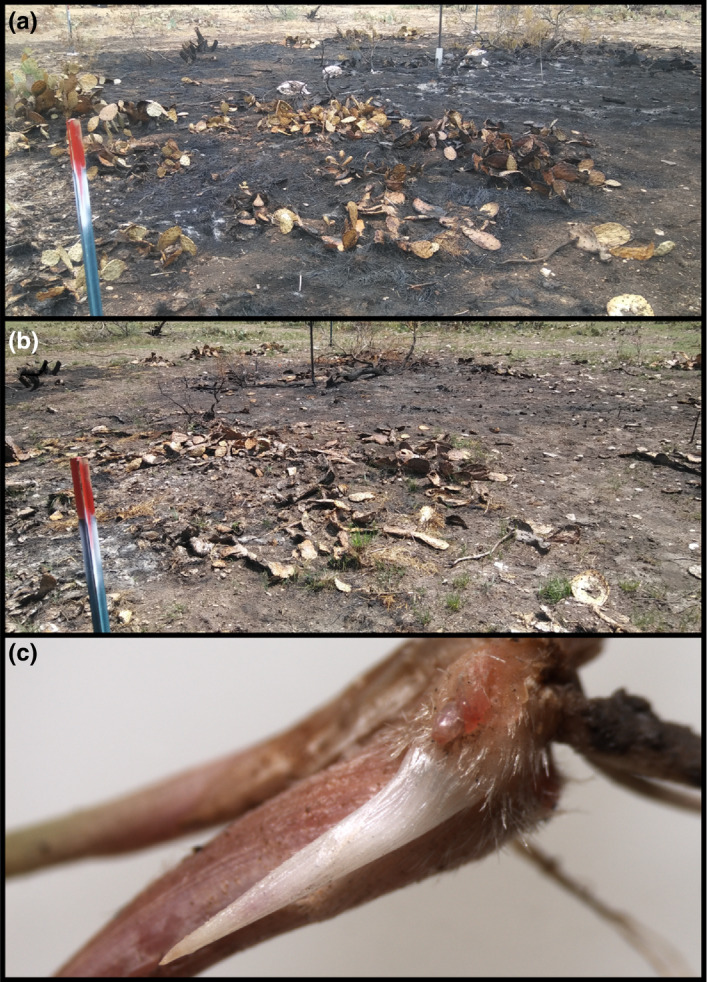
Picture of high‐energy plot immediately after treatment application (a), the same high‐energy plot 3 weeks post‐treatment (b), and a bud on a *Nassella leucotricha* tiller from a high‐energy plot post‐treatment (c)

Three weeks after the prescribed fire treatments, grasses in the high‐energy plots were beginning to resprout (Figure 4). However, all *H. belangeri* individuals except those in the high‐energy treatment showed regrowth. In the high‐energy treatment, 75% of the marked individuals failed to resprout (*p* < .001). Most of the individuals that failed to resprout were not completely consumed by the fire, though some were. Those that were not completely consumed still had intact belowground structures but few retained any aboveground tillers, live or dead. All marked *N. leucotricha* individuals resprouted in the low‐energy and high‐energy treatments following the fires.

## 4 DISCUSSION

### 4.1 Immediate effects of fire energy

The use of high‐energy fires as a management tool provides insight into the evaluation of the immediate effect of fire energy on grass bud bank dynamics. Although there are studies capturing the immediate bud bank dynamics of grasses following low‐ and moderate‐energy fires (Russell et al., 2013; Russell et al., 2015, 2019; Russell & Vermeire, 2015), this is the first look at the effect of high‐energy fires on bud responses. While soil has been shown to insulate belowground plant tissue from fire (Volland & Dell, 1981; Young, 1983), studies have shown a potential for soil heating to lethal temperatures, especially at shallower depths ( Balatsos, 1994; Campbell, 2016; Choczynska & Johnson, 2009; Kobziar et al., 2019; Peter, 1992). Increasing fuel consumption leads to increased fire energy (Kremens et al., 2012), increased soil surface heat fluxes and temperatures (Choczynska & Johnson, 2009), and greater soil heating (Bradstock & Auld, 1995). Therefore, high‐energy fires have a greater potential to impact grass bud metabolic activity and survival than low‐energy fires.

In this study, only high‐energy fires had a significant effect on immediate bud mortality for both species examined, with immediate increases in dead buds. The lack of significant bud mortality in our low‐energy plots corresponds to the results in Russell et al. (2015) even though their fuel loads were substantially lower than ours (0.15 vs. 0.6 kg/m^2^, respectively; Table 3). The fuel loads in our low‐energy treatment were lower, but closer, in approximation, to the 0.8 kg/m^2^ loadings that Haile (2011) found to result in a 50% probability of mortality for two different Great Plains grasses after heating trials. We can speculate that the relatively dry soils in our study reduced soil heating (Busse et al., 2013) and, in turn, reduced impacts on bud banks. The increase we saw in dead buds in our high‐energy plots is not necessarily indicative of the entirety of bud mortality. Some buds were likely consumed by fire and therefore were not captured in the count.

The increase in dead buds was much greater for *H. belangeri* than *N. leucotricha*. Even with this significant fire‐induced bud mortality, the absolute amount of bud death was relatively small. In the high‐energy treatment, approximately 24% of *N. leucotricha* buds and 34% of *H. belangeri* buds were dead following treatments. Even though these values seem relatively small, they may have differentially impacted our focal species and resulted in a difference in tiller reemergence following treatment. Therefore, high‐energy fires induce immediate bud mortality in these species, but the differences in the magnitude are likely mediated by growth form and photosynthetic pathway.

Fire energy also had a significant effect on the number of active buds for both *N. leucotricha* and *H. belangeri*. Both high‐ and low‐energy fires led to a decrease in active buds, but high‐energy fires led to a greater decrease for *N. leucotricha*. In contrast, *H. belangeri* experienced a similar decrease in active buds in both high‐ and low‐energy treatments, suggesting that fire energy was not as important for *H. belangeri* with regard to changes in bud activity.

Interestingly, dormant buds remained constant between pre‐ and post‐treatment sampling times for both species and across all treatments. Because we saw an increase in bud mortality and a decrease in active buds in our high‐energy treatment while dormant bud numbers remained constant, it is likely that the majority of the bud mortality we saw was from bud death in active buds. This lack of change in bud dormancy may be a product of the timing of our burns and the combination of heat and drought. Newly formed axillary buds often cycle through temporary transition stages of temporary growth or dormancy until developmental or environmental cues signal buds to undergo growth or fully become dormant (Devitt & Stafstrom, 1995; Shimizu‐Sato & Mori, 2001). When axillary buds are in these temporary transitions, environmental cues such as heat or drought can induce quiescence. This is likely what was seen in Russell et al. (2015), with one of their focal C_4_ species, *Bouteloua gracilis*, and may be why both of our species did not see an increase in bud activation and instead experienced little change in dormancy. This temporary bud strategy is particularly advantageous in areas, such as our semiarid site, that evolved under summer fire regimes and this temporary dormancy of buds allows plants to survive heat stress from increased summer fire intensities (Higgins, 1984; Umbanhowar, 1996).

With high‐energy fires during drought increasingly being applied to remove invasive shrubs, this study serves to assuage some fears in relation to extreme fires. High‐energy fires may cause immediate bud death, but it was not a large proportion of the available bud bank for either species. In addition, in the case of *N. leucotricha*, many dormant buds survived, and new tillers were produced a few weeks following treatment. With increased bud mortality, meristematic limitations can occur. Because the overall bud bank size plays a prominent role in plant population dynamics by buffering against disturbance (Benson et al., 2004; Dalgleish & Hartnett, 2009), a decrease in size can result in a decreased capacity to not only recover from disturbance but to also take advantage of the increased nutrient availability and light that often occurs following disturbances such as fire (Benson et al., 2004; Dalgleish & Hartnett, 2006). The fact that we see many *N. leucotricha* buds survive following these high‐energy fires may indicate that the local population of this grass is likely to persist and remain unchanged in the long‐term.

### 4.2 Growth form

Although we saw a direct effect of fire energy on bud mortality, the ratio of dead buds to active and dormant buds postfire was higher for *H. belangeri* than *N. leucotricha*. This result may be an effect of the relationship between fire energy and residence times and differential growth forms that influence bud depth within the soil profile. *Nassella leucotricha* had deeper buds, on average, than *H. belangeri*. Fuel consumption, fire energy, and residence times were substantially higher for high‐energy than low‐energy subplots (Tables 3 and 4).

This is important because soil is considered an effective insulator (Valettel et al., 1994). Choczynska and Johnson (2009) found that most of the temperature increase from fire in soils occurred in the first 1 cm of soil and dropped off steeply below 1 cm. They also found that lethal temperatures for 3 grass species in their study did not occur below the top 2 cm of soil, even with surface temperatures of 700°C sustained for 660 s. Since *N. leucotricha* buds were located at 1.8 cm below the soil surface on average, with many below the critical 2 cm depth described by Choczynska and Johnson (2009), while *H. belangeri* buds were located 0.5 cm below the soil surface on average (most within the top 0.55 cm), many more *H. belangeri* buds were likely within the lethal soil‐heating zone for high‐energy fires. Therefore, higher fuel consumption that results in greater fire energy and residence times is more important for grasses with shallower bud banks because soil heating is increased, and a larger portion of buds will fall within the lethal soil‐heating zone.

*Hilaria belangeri's* shallow bud depths are consistent with other stoloniferous species, particularly *Bouteloua dactyloides,* which was found to have growing points primarily at ground level or just below (Branson, 1953). Bunchgrasses have a range of growing points, with growing points extending above the soil to below the surface (Branson, 1953; Edmond & Hoveland, 1972), with one study estimating the mean depth of grass growth points in a tallgrass prairie being 3.2 ± 2.1 cm below the soil surface (Benson et al., 2004). Other studies showed patterns regarding bud distribution in the soil profile, bud death, and disturbance intensity (Klimešová & Klimeš, 2007; Vesk et al., 2004), suggesting bud depth may be an important determinant of grass survival following fires.

Previous studies found that bunchgrasses with caespitose growth forms are more susceptible to fire damage than other growth forms (Engle et al., 1998; Wright, 1971). In general, litter accumulates in the crown of caespitose grasses which increases fuel load at its center, leading to greater heat duration and dosage (Engle et al., 1998; Wright, 1971). This potentially increases bunchgrass susceptibility to fire by increasing bud mortality. This was not observed in our study. Instead, the stoloniferous species *H. belangeri* was more susceptible to fire damage than the caespitose species *N. leucotricha*.

This result is consistent with Russell et al. (2015) in which they found that *H. comata* did not sustain immediate bud mortality despite its bunchgrass form. Russell et al. (2015) reasoned that *H. comata's* coarse stems and dense plant crown prevented heat transfer to the buds and subsequent mortality. Size may also be a contributing factor in this study. Wright and Klemmedson (1965) suggest that, for some species, the size of the plant is important in determining the effect of fire on bunchgrasses, especially during the latter part of the summer. With our site being semiarid, our *N. leucotricha* individuals covered a small basal area (~7‐20 cm in diameter) and likely had lower accumulation of litter resulting in less heat duration at the center of the plant and less bud mortality. Overall, it is difficult to determine if growth form played a large role in bud response because of the number of herbaceous fuels added to the plot, potentially offsetting any effect of the grasses themselves on fire behavior.

### 4.3 Phenology

Fire season has been shown to directly affect bud activity, dormancy, and mortality for several grass species following moderate‐energy fires (Russell et al., 2015, 2019). Bud bank size and seasonal bud bank dynamics vary among species (Lehtilä, 2000; Zhang & Biswas, 2017) and have been shown to differ among grasses with different photosynthetic pathways (Ott & Hartnett, 2012). Therefore, another possible explanation for higher bud mortality in *H. belangeri* may be the timing of our burns. *Nassella leucotricha* is a C_3_, cool‐season grass while *H. belangeri* is a C_4_, warm‐season grass. These different functional groups have different phenological timing of increased bud activity or dormancy (Ott & Hartnett, 2012). Summer fires have been shown to favor C_3_ over C_4_ perennial grasses (Engle et al., 1998) because actively growing grasses are more easily damaged by fire than dormant grasses (Briske, 1991).

In Russell et al. (2015), moderate‐intensity summer fires had little impact on immediate bud activity or mortality in a C_3_ bunchgrass, *H. comata*. Although our low‐energy fires did not produce a significant increase in bud mortality, there was a decrease in active buds in *N. leucotricha*. In contrast, the sod‐forming C_4_ grass*, B. gracilis*, saw an immediate increase in bud activity following summer fires (Russell et al., 2015). Since total bud numbers did not change, there was likely a shift from dormant buds to active (Russell et al., 2015). We likely did not see this same shift because *H. belangeri* experienced a significant decrease in active buds with an increase in bud mortality. Given that total decrease in bud numbers, it is likely that active buds in both the low‐ and high‐energy plots experienced greater mortality than dormant buds.

All permanently marked *N. leucotricha* individuals produced new tillers in the high‐energy treatment. Although not all *H. belangeri* buds died in the high‐energy fires, very few individuals produce new tillers 3 weeks following fire despite increased precipitation following fires which led to many grass species, including *H. belangeri*, to resprout in the low‐energy plots. Since C_4_ grasses become dormant during the fall, early August fire likely induced dormancy earlier than usual due to increased stress from high‐energy fire and low water availability. We expect induced dormancy because bud activity and outgrowth are modulated by environmental conditions (Ott et al., 2019; Shimizu‐Sato & Mori, 2001). In fact, Russell et al. (2019) found that summer fire increased overwintering buds of C_4_ grasses. This is most likely the reason resprouting did not occur in our high‐energy treatments. However, a longer‐term examination of fire seasonality effects on bud dynamics is warranted for corroboration (Hiers, 2019).

Differences between C_3_ and C_4_ grass responses to fire energy manifest over longer time frames since overall bud numbers, overwintering strategies, and seasonal patterns of bud activity all drive long‐term grass responses to fire and have been shown to differ between C_3_ and C_4_ grasses (Ott & Hartnett, 2012; Russell et al., 2015). So, although functional group has a large influence on phenological patterns of bud growth and dormancy (Ott & Hartnett, 2012; Russell et al., 2015), and therefore grass response to fire energy, the difference in photosynthetic pathway seems less important for immediate bud response to fire energy than growth form in our study.

Despite indications of immediate loss of vegetative reproduction and potential mortality of individuals, these results may not necessarily translate to loss of biomass of these species over longer time periods. Studies of long‐term grazing have shown that bud numbers for *H. belangeri* were significantly greater in grazed than ungrazed communities due to increases in plant density (Hendrickson & Briske, 1997). Hendrickson and Briske (1997) demonstrated that long‐term effects of herbivory, and likely other disturbances, are predominantly expressed at the population level rather than at the individual or tiller level. In other semiarid savanna ecosystems, a reduction in neighborhood density of perennial grasses can increase the long‐term survival and productivity of surviving individuals (Zimmerman et al., 2010). In addition, increases in nutrient availability and light can offset direct loss of buds, allowing for increased growth and reproduction in years following fire (Dalgleish & Hartnett, 2008; Russell & Vermeire, 2015; Tomlinson & O'Connor, 2004). These studies point to the likelihood that *H. belangeri* should recover in the future despite indications of decreased vegetative reproductivity and possible meristem limitations.

Also, given that our study site tends to have patchy herbaceous cover, pockets of grasses would be protected from high‐intensity fires under more natural conditions (i.e., without added fuel). We also expect to see that, due to spatial heterogeneity, stoloniferous and rhizomatous grasses in areas not impacted by high‐intensity fires will colonize areas that were affected. Additionally, recruitment from the seedbank will likely impact the colonization of areas but to what extent is a potential future area of study. Although we saw greater bud death in *H. belangeri* and little regrowth, long‐term studies indicate that high‐intensity fires do not lead to legacy changes in the herbaceous understory. As such, we expect that *H. belangeri* will recover in the next few growing seasons (Taylor et al., 2012).

## 5 CONCLUSIONS

Most grass regrowth occurs primarily via a belowground bank of axillary buds (Latzel et al., 2008; Ott et al., 2019; Vítová et al., 2017). It is therefore necessary to understand bud bank dynamics to predict grass population and community responses to disturbances such as fire (Benson & Hartnett, 2006; Dalgleish & Hartnett, 2009). In this study, we saw a significant increase in bud mortality in both our species in the high‐energy treatment; however, this bud mortality was greater in *H. belangeri* and monitored individuals failed to resprout 3 weeks following treatment application. Our immediate bud responses are most likely the result of contrasts in the phenology and growth forms of our two grass species, which suggests the need for managers to consider both in predicting grass survival following high‐energy fires during low water availability.

## CONFLICT OF INTEREST

None declared.

## AUTHOR CONTRIBUTIONS

**Quinn A. Hiers:** Formal analysis (equal); Methodology (equal); Visualization (lead); Writing‐original draft (lead); Writing‐review & editing (equal). **Morgan L. Treadwell:** Conceptualization (equal); Formal analysis (equal); Methodology (equal); Validation (equal); Writing‐original draft (supporting); Writing‐review & editing (equal). **Matthew B. Dickinson:** Conceptualization (equal); Methodology (equal); Writing‐review & editing (equal). **Kathleen L. Kavanagh:** Conceptualization (equal); Methodology (equal); Writing‐review & editing (equal). **Alexandra G. Lodge:** Conceptualization (equal); Methodology (equal); Writing‐review & editing (equal). **Heath D. Starns:** Methodology (equal); Writing‐review & editing (equal). **Doug R. Tolleson:** Methodology (equal); Writing‐review & editing (equal). **Dirac Twidwell:** Conceptualization (equal); Writing‐review & editing (equal). **Carissa L. Wonkka:** Conceptualization (equal); Methodology (equal); Writing‐original draft (supporting); Writing‐review & editing (equal). **William E. Rogers:** Conceptualization (equal); Funding acquisition (lead); Methodology (equal); Supervision (lead); Writing‐original draft (supporting); Writing‐review & editing (equal).

## Supporting information

Fig S1

## Data Availability

Data are archived in the Dryad Digital Repository (https://doi.org/10.5061/dryad.kprr4xh48).
