# Supplementary material for: Grass bud responses to fire in a semiarid savanna system
Source: Ecol Evol. 2021 Apr 7;11(11):6620–33. doi: 10.1002/ece3.7516 (PMC8207346; doi:10.1002/ece3.7516)
Supplement: Supplementary file 1 — Fig S1 [file ECE3-11-6620-s001.docx]

- 1. Supplemental Information


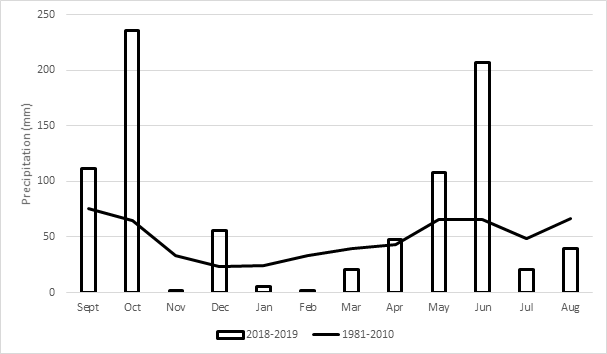


**Figure S1**: Study site mean precipitation (mm), averaged over the periods 1981-2010 and 2018-2019. Data retrieved from on-site weather station for 2018-2019. Averages for 1981-2010 retrieved from National Climatic Data Center for Sonora, Texas. All fire treatment plots were burned between July 30^th^ and August 4^th^ 2018.
